# Increasing Trends of Polypharmacy and Potentially Inappropriate Medication Use in Older Lung Cancer Patients in China: A Repeated Cross-Sectional Study

**DOI:** 10.3389/fphar.2022.935764

**Published:** 2022-07-18

**Authors:** Fangyuan Tian, Zhaoyan Chen, Xi Chen, Mengnan Zhao

**Affiliations:** ^1^ Department of Pharmacy, West China Hospital, Sichuan University, Chengdu, China; ^2^ Department of Epidemiology and Health Statistics, West China School of Public Health and West China Fourth Hospital, Sichuan University, Chengdu, China; ^3^ Department of Integrated Care Management Center, West China Hospital, Sichuan University, Chengdu, China

**Keywords:** polypharmacy, potentially inappropriate medication, lung cancer, older, outpatient

## Abstract

**Objectives:** Polypharmacy and potentially inappropriate medication (PIM) use are frequent in older lung cancer patients. This study aimed to examine the trends of polypharmacy and PIM use and explore risk factors for PIM use based on the 2019 Beers criteria in older Chinese lung cancer outpatients with multimorbidity.

**Methods:** A repeated cross-sectional study was conducted using electronic medical data consisting of the prescriptions of older lung cancer outpatients in China from January 2016 to December 2018. Polypharmacy was defined as the use of five or more medications. The 2019 Beers criteria were used to evaluate the PIM use of older cancer outpatients (age ≥65 years), and multivariate logistic regression was used to identify the risk factors for PIM use.

**Results:** A total of 3,286 older lung cancer outpatients and their prescriptions were included in the study. The prevalence of polypharmacy was 14.27% in 2016, 16.55% in 2017, and 18.04% in 2018. The prevalence of PIM use, according to the 2019 Beers criteria, was 31.94% in 2016, 35.78% in 2017, and 42.67% in 2018. The two most frequently used PIMs in older lung cancer outpatients were estazolam and tramadol. The logistic regression demonstrated that age 75 to 79, polypharmacy, irrational use of drugs, and lung cancer accompanied by sleep disorders, anxiety or depression, or pain were positively associated with PIM use in older lung cancer outpatients.

**Conclusion:** The prevalence of polypharmacy and PIM use in older lung cancer outpatients with multimorbidity was high in China, and polypharmacy and PIM use increased over time. Further research on interventions rationing PIM use in the older lung cancer patient population is needed.

## Introduction

Lung cancer is the leading cause of cancer death worldwide, accounting for nearly 1.80 million deaths and causing 18% of total cancer deaths in 2020 (Global Cancer Observatory, 2020; WHO, 2022). Older age is associated with cancer development due to biological factors that include DNA damage over time and shortened telomeres. As the population continues to age, the incidence of lung cancer in older patients is expected to further increase in the coming years (Decoster and Schallier, 2019). Approximately 37% of lung cancer cases occur in individuals over 75 years old. Accordingly, the median age at lung cancer diagnosis is 70 years old for both men and women (Torre et al., 2016). The majority of older lung cancer patients have comorbid chronic diseases and must take multiple medications (Grose et al., 2014; Nilsson et al., 2017; Ding et al., 2020). However, increased number of drug-related problems was associated with age-induced alternations in pharmacokinetics (PK) and pharmacodynamics (PD). Lung cancer also effects pharmacokinetics and pharmacodynamics and occurs more frequently in the elderly. Therefore, lung cancer patients who are elderly are more prone to experience adverse drug events. In addition, previous published studies have confirmed that cancer patients are easily exposed to a higher risk of polypharmacy and inappropriate medication use (Wildiers et al., 2014; Koczwara et al., 2022).

Polypharmacy (defined as the use of more than five medicines) is associated with the prescription of inappropriate medications, and extensive studies have demonstrated the link between polypharmacy and negative outcomes (Maddison et al., 2011; Weng et al., 2013; LeBlanc et al., 2015). Potentially inappropriate medication (PIM) use was firstly proposed in 1991 because PIM use brought a series of drug-related problems, such as adverse drug events, hospitalization, and disability, defined as the use of medications that should be avoided, especially when evidence is insufficient or alternative medicines are available (Hyttinen et al., 2016; Muhlack et al., 2017; Wallace et al., 2017).

Some previous reports have examined the trends of polypharmacy and PIM use in older patients (Davidoff et al., 2015; Moriarty et al., 2015; Muhlack et al., 2018). Approximately half of all cancer care is delivered in an outpatient treatment setting (Maleki et al., 2022). It is necessary to investigate the polypharmacy and PIM use in older lung cancer outpatients. However, no study has specifically reported on the trends of polypharmacy and PIM use in older lung cancer outpatients, and the risk factors for PIM use according to the 2019 Beers criteria in older Chinese lung cancer patients are unclear. Therefore, in this study, we extracted data on the prescriptions of older lung cancer outpatients treated at tertiary hospitals in Chengdu, China over 3 years. The trends of the prevalence of polypharmacy and PIM use were calculated, and PIMs were screened based on the 2019 Beers criteria. The risk factors for PIM use were explored. Ideally, this study will provide useful data for follow-up research.

## Methods

### Setting and Sample

A repeated cross-sectional study was performed to examine the trends of polypharmacy and PIM use among older lung cancer (histology: non-small-cell lung cancer, small cell lung cancer, unspecified lung cancer; stage: American Joint Commission on Cancer 8th Edition (AJCC) stage I-III) outpatients with multimorbidity that might receive chemotherapy and chronic disease treatment in tertiary hospitals in Chengdu, a capital city in southwest China. The prescriptions of older (aged ≥65) lung cancer outpatients with multimorbidity (cancer with other diseases) were cluster sampled from a cooperative hospital prescription analysis project led by the Chinese Pharmaceutical Association. In this study, cluster sampling was used to randomly select nine hospitals from all tertiary hospitals in Chengdu between 1 January 2016 and 31 December 2018, and then the older lung cancer outpatient prescriptions were selected from all departments of the selected hospitals as the survey samples. Multimorbidity of patients was determined by the numbers of diagnosis in medical record. All data were retrospectively collected without any possibility of individual identification.

### Data Collection

In this repeated cross-sectional study, we included older adults with lung cancer attending outpatient department at tertiary care hospitals in Chengdu from 1 January 2016 to 31 December 2018; thus, the prescriptions of 1,002, 1,009, and 1,275 older lung cancer outpatients were included from 2016, 2017, and 2018, respectively. The data were collected by diagnosis type as follows: 1) basic information (region, prescription code, and department source); 2) patient characteristics (age, sex, and diagnosis); and 3) medication characteristics (generic name, trade name, drug specifications, dosage form, administration route, number of prescriptions, prescription expenditure, and frequency of administration). The criteria in the count of prescribed medications are as follows: 1) duration of prescription (≤1 month); 2) route of administration (oral medications, injection medications, topical medications, inhaler, etc.); 3) medications directly related to treatment for lung cancer were counted as concomitant medications (such as oral tyrosine kinase inhibitor or antiemetic for chemo); 4) Chinese traditional herbal medications were not included.

### Evaluation Criteria

The 2019 Beers criteria (By the 2019 American Geriatrics Society Beers Criteria^®^ Update Expert Panel, 2019) were used to evaluate PIM use in older lung cancer outpatients who were not receiving palliative care or hospice service. The comments about the rationality of prescription were made according to the Chinese Prescription Administrative Policy. The Chinese Prescription Administrative Policy need pharmacists to evaluate the standardization of prescription and the suitability of clinical use of drugs (medication indications, drug selection, route of administration, usage and dosage, drug interaction, incompatibility, etc.) according to relevant regulations, finding existing or potential problems, formulating and implementing intervention and improvement measures to promote the rational application of clinical drugs. Irrational prescriptions were classified as nonstandard prescriptions, inappropriate prescriptions, and supernormal prescriptions referring to medication without indications. Any inconsistencies between the two researchers were reviewed by a third professional and then resolved through collective discussion.

### Statistical Analysis

Categorical data are presented according to frequency, and the χ^2^ test was used to compare categorical variables between groups. Continuous data that were normally distributed are expressed as the mean ± standard deviation (SD), and continuous data that were not normally distributed are expressed as the median (M) and the interquartile range (IQR). Participant sex was categorized as male or female. Age was categorized into four groups: 65–69, 70–74, 75–79, and ≥80 years old, and the number of diseases was divided into three groups: two, three to four, and five or more chronic conditions. For the descriptive analysis, medication use was divided into two strata: the use of one to four medications and the use of five or more medications. Prescriptions were further categorized as rational or irrational. The prescription expenditure was divided into three groups: <500 Chinese yuan, 500–1,000 Chinese yuan, and >1,000 Chinese yuan. Five chronic diseases (sleep disorders, anxiety or depression, pain, pulmonary infection, and chronic obstructive pulmonary disease) were also analyzed. The associations between the risk factors and PIM use (non-PIM use = 0, PIM use = 1) were assessed with a multivariate logistic regression analysis. Statistical analyses were conducted using SPSS version 26.0 (IBM Corp., Armonk, NY). We constructed three models: Model 1 (logistic regression with no adjustment), Model 2 (adjusted for year), and Model 3 (adjusted for year, sex and age). The results are presented as odds ratios (ORs) and 95% confidence intervals (CIs), and *p* < 0.05 was considered to be statistically significant.

### Ethics Approval

This study protocol was approved by the Sichuan University West China Hospital Research Ethics Board (2020/651).

## Results

### Basic Patient Characteristics

A total of 3,286 older lung cancer outpatients were included in this study, 55.90% (*n* = 1,837) of which were male. The median age was 72 (IQR: 68, 76) years old, and age ranged from 65 to 94 years old; the oldest (≥80 years of age) cancer patients accounted for 11.63% (*n* = 1,153) of the sample. The median number of medical diagnoses was 3 (IQR: 2, 4). The median number of prescriptions was 2 (IQR: 1, 4), and 16.43% (*n* = 540) of older lung cancer outpatients had polypharmacy. The prevalence of rational prescriptions was 92.64% (*n* = 3,044), 7.36% (*n* = 242) had irrational prescription. The characteristics of 242 participants were 60.74% (*n* = 147) of which were male, the median age was 72 years older and 42.98% (*n* = 104) of patients had polypharmacy. The median prescription expenditure was 517.90 (IQR: 189.50, 1309.47) Chinese yuan (CNY). In this study, 19.32% (*n* = 635) of the lung cancer patients had sleep disorders, 3.13% (*n* = 103) had anxiety or depression, 24.01% (*n* = 789) had pain, 11.56% (*n* = 380) had pulmonary infections, and 5.11% (*n* = 168) had chronic obstructive pulmonary disease (COPD). The basic patient characteristics are provided in Table 1.

**TABLE 1:** Basic characteristics of older lung cancer outpatients.

Characteristic	Total	2016 (N = 1,002)			2017 (N = 1,009)			2018 (N = 1,275)		
		PIM Group	Non-PIM Group	*p* Value	PIM Group	Non-PIM Group	*p* Value	PIM Group	Non-PIM Group	*p* Value
N (%)	3,286	320 (31.94)	682 (68.06)		361 (35.78)	648 (64.22)		544 (42.67)	731 (57.33)	
Sex, n (%)				0.434			0.626			0.677
Male	1,837 (55.90)	176 (55.00)	393 (57.62)		206 (57.06)	380 (58.64)		310 (56.99)	408 (55.81)	
Female	1,449 (44.10)	144 (45.00)	289 (42.38)		155 (42.94)	268 (41.36)		234 (43.01)	323 (44.19)	
Age, years (IQR), n (%)	72 (68, 76)	72 (68, 76)		0.561	71 (68, 76)		<0.001	72 (68, 76)		0.199
65–69	1,222 (37.19)	123 (38.44)	251 (36.80)		138 (38.23)	248 (38.27)		183 (33.64)	279 (38.17)	
70–74	959 (29.18)	87 (27.19)	209 (30.65)		86 (23.82)	203 (31.33)		157 (28.86)	217 (29.69)	
75–79	723 (22.00)	71 (22.19)	154 (22.58)		101 (27.98)	125 (19.29)		124 (22.79)	148 (20.25)	
≥80	382 (11.63)	39 (12.19)	68 (9.97)		36 (9.97)	72 (11.11)		80 (14.71)	87 (11.90)	
No. of diseases [IQR]	3 [2, 4]	3 [2, 4]		0.013	3 [2, 4]		0.072	3 [2, 5]		0.275
2	1,061 (32.29)	100 (31.25)	223 (32.70)		128 (35.46)	204 (31.48)		160 (29.41)	246 (33.65)	
3–4	1,492 (45.40)	137 (42.81)	336 (49.27)		153 (42.38)	323 (49.85)		240 (44.12)	303 (41.45)	
≥5	733 (22.31)	83 (25.94)	123 (18.04)		80 (22.16)	121 (18.67)		144 (26.47)	182 (24.90)	
No. of medications [IQR], n (%)	2 [1, 4]	2 [1, 3]		0.001	2 [1, 4]		<0.001	2 [2, 4]		<0.001
1–4	2,746 (83.57)	257 (80.31)	602 (88.27)		279 (77.29)	563 (86.88)		439 (80.70)	606 (82.90)	
≥5	540 (16.43)	63 (19.69)	80 (11.73)		82 (22.71)	85 (13.12)		105 (19.30)	125 (17.10)	
No. of rational prescriptions, n (%)				<0.001			<0.001			0.135
rational prescriptions	3,044 (92.64)	263 (82.19)	651 (95.45)		315 (87.26)	621 (95.83)		503 (92.46)	691 (94.53)	
irrational prescriptions	242 (7.36)	57 (17.81)	31 (4.55)		46 (12.74)	27 (4.17)		41 (7.54)	40 (5.47)	
Prescription expenditure [IQR], n (%)	517.90 (189.50, 1309.47)	597.54 (191.68, 1483.87)		<0.001	521.44 (218.60, 1281.72)		<0.001	487.29 (160.00, 1172.25)		<0.001
<500 CNY	1,597 (48.60)	189 (59.06)	268 (39.30)		220 (60.94)	270 (41.67)		318 (58.46)	332 (45.42)	
500–1,000 CNY	637 (19.39)	43 (13.44)	143 (20.97)		51 (14.13)	147 (22.69)		94 (17.28)	159 (21.75)	
>1,000 CNY	1,052 (32.01)	88 (27.50)	271 (39.74)		90 (24.93)	231 (35.65)		132 (24.26)	240 (32.83)	
Cancer type				0.574			0.007			0.591
Unspecified lung cancer	1,656 (50.40)	149 (46.56)	298 (43.70)		194 (53.74)	304 (46.91)		310 (56.99)	401 (54.86)	
NSCLC	1,503 (45.74)	159 (49.69)	351 (51.47)		161 (44.60)	311 (47.99)		214 (39.34)	307 (42.00)	
SCLC	127 (3.86)	12 (3.75)	33 (4.84)		6 (1.66)	33 (5.09)		20 (3.68)	23 (3.15)	
Type of chronic disease, n (%)										
Sleep disorder	635 (19.32)	101 (31.56)	59 (8.65)	<0.001	147 (40.72)	53 (8.18)	<0.001	229 (42.10)	46 (6.29)	<0.001
Anxiety or depression	103 (3.13)	21 (6.56)	9 (1.32)	<0.001	14 (3.88)	10 (1.54)	0.020	37 (6.80)	12 (1.64)	<0.001
Pain	789 (24.01)	131 (40.94)	103 (15.10)	<0.001	149 (41.27)	90 (13.89)	<0.001	237 (43.56)	88 (12.04)	<0.001
Pulmonary infection	380 (11.56)	48 (15.00)	95 (13.93)	0.652	42 (11.63)	63 (9.72)	0.340	42 (7.72)	90 (12.32)	0.008
COPD	168 (5.11)	10 (3.13)	29 (4.25)	0.390	10 (2.77)	37 (5.71)	0.034	29 (5.33)	53 (7.25)	0.167

PIM, potentially inappropriate medication; IQR, interquartile range; CNY, chinese yuan; NSCLC, non-small-cell lung cancer; SCLC, small cell lung cancer; COPD, chronic obstructive pulmonary disease.

### Trends in Older Lung Cancer Outpatients With Multimorbidity

**FIGURE 1:**
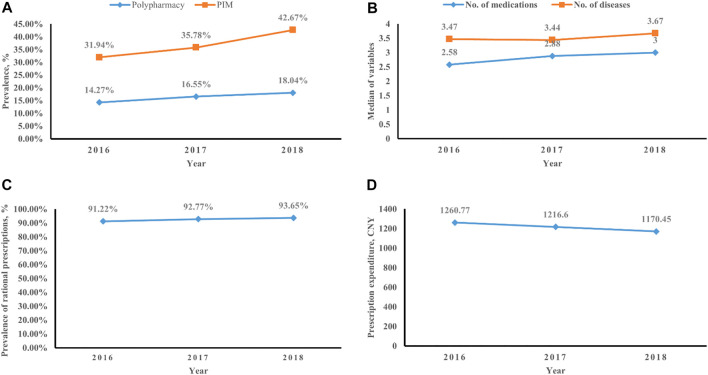
Trends in older lung cancer outpatients with multimorbidity. **(A)** Prevalence of polypharmacy and PIM use. **(B)** Number of medications and diseases. **(C)** Rate of rational prescriptions. **(D)** Prescription expenditure.

There were 1,002, 1,009, and 1,275 older lung cancer outpatients with prescriptions included in 2016, 2017, and 2018, respectively. The prevalence of polypharmacy increased from 14.27% (*n* = 143) in 2016 to 18.04% (*n* = 230) in 2018. The prevalence of PIM use increased from 31.94% (*n* = 320) to 42.67% (*n* = 544) over the 3 years. The number of medications and diseases showed an increasing trend from 2016 to 2018. The prevalence of rational prescriptions increased from 91.22% (*n* = 914) in 2016 to 93.65% (*n* = 1194) in 2018, but the average prescription expenditure showed a decreasing trend from 1,260.77 CNY per prescription in 2016 to 1,170.45 CNY per prescription in 2018 (Figure 1).

### Prevalence of PIMs and the Most Frequent PIMs Over the Three Years

Among the 1,002 older lung cancer outpatients with prescriptions in 2016, 320 (31.94%) outpatients were identified to have at least one PIM, and a total of 428 PIMs were detected according to the 2019 Beers criteria. Of the patients with PIM prescriptions, 80.00% received one PIM, 15.31% received two PIMs, and 4.69% received at least three PIMs according to the criteria (Table 2). Overall, estazolam, tramadol, and megestrol were the most used PIMs according to the 2019 Beers criteria, at 18.60%, 13.44, and 12.92%, respectively (Table 3).

**TABLE 2:** The number of PIMs used by older lung cancer outpatients.

Characteristic	2016	2017	2018
PIM prescription	320	361	544
PIMs, n (%)	428	480	723
1PIM	256 (80.00)	297 (82.27)	439 (80.70)
2 PIMs	49 (15.31)	41 (11.36)	66 (12.13)
≥3 PIMs	15 (4.69)	23 (6.37)	39 (7.17)

PIM, potentially inappropriate medication.

**TABLE 3:** The top five PIMs used by older lung cancer outpatients.

Rank	2016	N = 387 (%)	2017	N = 433 (%)	2018	N = 664 (%)
1	Estazolam	72 (18.60)	Estazolam	92 (21.25)	Estazolam	149 (22.44)
2	Tramadol	52 (13.44)	Tramadol	68 (15.70)	Tramadol	116 (17.47)
3	Megestrol	50 (12.92)	Megestrol	46 (10.62)	Ibuprofen	62 (9.34)
4	Ibuprofen	33 (8.53)	Ibuprofen	36 (8.31)	Alprazolam	51 (7.68)
5	Hydrochlorothiazide	29 (7.49)	Hydrochlorothiazide	34 (7.85)	Hydrochlorothiazide	51 (7.68)

Among the 1,009 older lung cancer outpatients with prescriptions in 2017, 361 (35.78%) outpatients were identified to have at least one PIM, and a total of 480 PIMs were detected by the 2019 Beers criteria. Of the patients with PIM prescriptions, 82.27% received one PIM, 11.36% received two PIMs, and 6.37% received at least three PIMs according to the criteria (Table 2). Overall, estazolam, tramadol, and megestrol were the most used PIMs according to the 2019 Beers criteria, at 21.25%, 15.70, and 10.62%, respectively (Table 3).

Among the 1,275 older lung cancer outpatients with prescriptions in 2018, 544 (42.67%) outpatients were identified to have at least one PIM, and a total of 723 PIMs were detected according to the 2019 Beers criteria. Of the patients with PIM prescriptions, 80.70% received one PIM, 12.13% received two PIMs, and 7.17% received at least three PIMs according to the criteria (Table 2). Overall, estazolam, tramadol, and ibuprofen were the most used PIMs according to the 2019 Beers criteria, at 22.44%, 17.47, and 9.34%, respectively (Table 3).

### Risk Factors for PIM Use

PIM use, based on the 2019 Beers criteria, was the dependent variable in the logistic regression analysis (non-PIM use = 0, PIM use = 1). The logistic regression analysis indicated that age 75–79 (OR: 1.276 in Model 1, OR: 1.273 in Model 2), polypharmacy (OR: 2.587 in Model 1, OR: 2.672 in Model 2, OR: 2.678 in Model 3), and the irrational use of drugs (OR: 2.146 in Model 1, OR: 2.082 in Model 2, OR: 2.078 in Model 3) were positively associated with PIM use in older lung cancer outpatients. Older lung cancer patients with sleep disorders (OR: 11.408 in Model 1, OR: 11.433 in Model 2, OR: 11.158 in Model 3), anxiety or depression (OR: 5.079 in Model 1, OR: 5.135 in Model 2, OR: 4.834 in Model 3), and pain (OR: 7.021 in Model 1, OR: 7.047 in Model 2, OR: 6.884 in Model 3) were more likely to have PIM prescriptions (Table 4).

**TABLE 4:** Multivariate logistic regression analysis of factors associated with PIM use.

Model 1				Model 2				Model 3			
Characteristic	OR	95% CI	*p* Value	Characteristics	OR	95% CI	*p* Value	Characteristics	OR	95% CI	*p* Value
Year											
2016	References										
2017	1.069	0.855–1.335	0.559								
2018	1.432	1.161–1.766	0.001								
Sex				Sex							
female	References			female	References						
male	1.176	0.985–1.404	0.074	male	1.172	0.982–1.398	0.079				
Age, y				Age, y							
65–69	References			65–69	References						
70–74	0.793	0.639–0.983	0.034	70–74	0.796	0.642–0.987	0.038				
75–79	1.276	1.016–1.602	0.036	75–79	1.273	1.014–1.597	0.037				
≥80	1.040	1.146–1.525	0.790	≥80	1.056	0.793–1.407	0.707				
No. of diseases				No. of diseases				No. of diseases			
2	References			2	References			2	References		
3–4	0.868	0.710–1.061	0.166	3–4	0.862	0.706–1.053	0.145	3–4	0.859	0.703–1.048	0.135
≥5	0.709	0.545–0.922	0.01	≥5	0.726	0.559–0.943	0.016	≥5	0.749	0.577–0.971	0.029
No. of medications				No. of medications				No. of medications			
1–4	References			1–4	References			1–4	References		
≥5	2.587	1.988–3.367	<0.001	≥5	2.672	2.054–3.475	<0.001	≥5	2.678	2.063–3.478	<0.001
No. of rational prescriptions				No. of rational prescriptions				No. of rational prescriptions			
Rational prescriptions	References			Rational prescriptions	References			Rational prescriptions	References		
Irrational prescriptions	2.146	1.548–2.977	<0.001	Irrational prescriptions	2.082	1.500–2.890	<0.001	Irrational prescriptions	2.078	1.498–2.822	<0.001
Prescription expenditure				Prescription expenditure				Prescription expenditure			
<500 CNY	References			<500 CNY	References			<500 CNY	References		
500–1,000 CNY	0.486	0.383–0.617	<0.001	500–1,000 CNY	0.483	0.380–0.612	<0.001	500–1,000 CNY	0.484	0.382–0.614	<0.001
>1,000 CNY	0.419	0.336–0.521	<0.001	>1,000 CNY	0.404	0.324–0.503	<0.001	>1,000 CNY	0.404	0.325–0.503	<0.001
Type of chronic disease				Type of chronic disease				Type of chronic disease			
Sleep disorder	11.408	9.061–14.362	<0.001	Sleep disorder	11.433	9.091–14.378	<0.001	Sleep disorder	11.158	8.901–13.987	<0.001
Anxiety or depression	5.079	2.987–8.637	<0.001	Anxiety or depression	5.135	3.032–8.695	<0.001	Anxiety or depression	4.834	2.866–8.152	<0.001
Pain	7.021	5.753–8.569	<0.001	Pain	7.047	5.776–8.596	<0.001	Pain	6.884	5.653–8.383	<0.001
Pulmonary infection	0.831	0.629–1.097	0.190	Pulmonary infection	0.811	0.615–1.069	0.137	Pulmonary infection	0.817	0.621–1.076	0.150
COPD	0.772	0.511–1.168	0.221	COPD	0.806	0.534–1.214	0.302	COPD	0.840	0.559–1.261	0.840

PIM, potentially inappropriate medication; IQR, interquartile range; CNY, Chinese yuan; COPD, chronic obstructive pulmonary disease.

Model 1: Multivariate logistic regression analysis of factors associated with PIM use in older lung cancer outpatients.

Model 2: Multivariate logistic regression analysis of factors associated with PIM use in older lung cancer outpatients adjusted by year.

Model 3: Multivariate logistic regression analysis of factors associated with PIM use in older lung cancer outpatients adjusted by year, sex, and age.

## Discussion

To the best of our knowledge, this is the first study to assess the trends of polypharmacy and PIM use in older Chinese lung cancer outpatients with multimorbidity. Previous studies based on national representative surveys have shown an alarming increase in the polypharmacy trends in the United States (from 8.2% in 1999 to 15% in 2012; Kantor et al., 2015), Sweden (from 16.9% in 2006 to 19.0% in 2014; Zhang et al., 2020), and France (from 44.9% in 2011 to 47.8% in 2019; Drusch et al., 2021), which are consistent with regional register-based studies on this period in the United Kingdom (a polypharmacy increase from 11.2% in 1995 to 22.8% in 2010; Guthrie et al., 2015) and those using the University Groningen IADB.nl prescription database in the Netherlands (showing an increase from 56.5% in 2012 to 58.2% in 2016; Oktora et al., 2021). Subjective measures, such as sleep diaries and anxiety and depression screening scales, also assist the diagnosis. As the diagnosis is determined, the use of drugs may be further increased (Hita-Yañez et al., 2013). In our study, the number of medications and diseases showed an increasing trend from 2016 to 2018. Therefore, the prevalence of polypharmacy increased in our study. Some studies in Europe and the United States have reported a decrease in the prevalence of PIMs (Ble et al., 2012; Hovstadius et al., 2014; Davidoff et al., 2015; Muhlack et al., 2018). With the popularization of Beers criteria, clinicians pay more attention to PIMs use in older patients; however, due to the increase of chronic diseases, the number of medications increased. This may be the reason that studies in the US indicate increased polypharmacy yet decrease in prevalence of PIMs use. However, one study in Ireland showed that the prevalence of PIM use rose from 32.6% in 1997 to 37.3% in 2012 (Moriarty et al., 2015). Our previous study showed an increasing trend of PIM use in older inpatients, from 71.17% in 2016 to 73.39% in 2018 (Tian et al., 2021). These results were similar to those of our present study, in which an increased prevalence of PIM use was observed in an older lung cancer patient population. Because polypharmacy is associated with an increased risk of inappropriate prescriptions, the prevalence of polypharmacy and PIM use in our studies were related, showing an increasing trend.

Our study was the first repeated cross-sectional study on the prevalence and risk factors for PIM use in older Chinese lung cancer outpatients. A United States study reported that the monthly prevalence of any PIM prior to cancer diagnosis was similar across all three cancer cohorts (breast cancer, colon cancer, and lung cancer), hovering between 37 and 40%, whereas PIM prevalence sharply increased in the first few months following the lung cancer (stage I–II) diagnosis. This may be when the lung cancer is diagnosed, the anti-emetics, antispasmodic drugs, and hydrochlorothiazide were usually used (Lund et al., 2018). The prevalence of PIM use in older lung cancer patients was higher than that in patients with the other two cancers according to the 2012 Beers criteria. The prevalence of PIM use, according to the 2019 Beers criteria, in our study was 37.28% over 3 years, which was higher than that reported in another study on the prevalence of PIM use among older Chinese cancer outpatients which were outside of palliative care and hospice service (32.65%; Tian et al., 2022a). Our previous study found that lung cancer was positively associated with PIM use in older cancer outpatients, which explains the higher prevalence of PIM use in this study. It is of great significance to study the high-risk population of PIMs use and provide targeted drug intervention for the follow-up. The prevalence of PIM use in our study was slightly higher than that the older advanced NSCLC patients who underwent epidermal growth factor receptor tyrosine kinase inhibitor in Japan, with a prevalence of 31.9% (Hakozaki et al., 2021), and lower than that in older NSCLC and SCLC patients at the end of life in the Netherlands, at 45% (Ham et al., 2021). The high prevalence of PIM use in these end-of-life patients is explained by the fact that these patients are usually in serious condition, both physically and mentally, and are thus highly willing to take more medications. Another potential reason for this difference is that the adverse outcomes in older lung cancer patients at the end of life are highly associated with PIM use; the poor clinical outcome of these patients further aggravates the prevalence of PIM use (Mohamed et al., 2020; Chen et al., 2021).

In our present study, the two most frequent PIMs in older Chinese lung cancer outpatients according to the 2019 Beers criteria were estazolam and tramadol over the 3 years. Sleep disorders can be both complex and common in older age, although reported prevalence varies (Hishikawa et al., 2017; Patel et al., 2018). Although research on the causal effect of sleep disorders on lung cancer incidence is still lacking, many lung cancer patients were also diagnosed with sleep disorders or pain in our study. Use of estazolam, a benzodiazepine, increases the risk of falls in older adults and co-prescribing of opioids exponentiates this risk (Niznik et al., 2022). Meanwhile, long-term use of benzodiazepines will increase the risk of respiratory depression and overdose with administration of benzodiazepine in older patients with sleep disorders. Pain is the most common symptom that occurs in 40% of lung cancer patients (Iwase et al., 2015), which can arise as a result of local effects (i.e., hemorrhaging into the tumor, obstruction/perforation of the lungs) or anti-cancer treatments, such as chemotherapy (Tang et al., 2021). The treatment of cancer pain mostly utilizes the three-step “ladder” treatment principle proposed by the World Health Organization; tramadol is commonly used for mild and moderate pain (Tian et al., 2022b). Although the analgesic effect of tramadol is good, its side effects induce syndrome of inappropriate antidiuretic hormone secretion (SIADH) or hyponatremia, and these risks are higher in older adults, which limits the clinical application of this medication (Li et al., 2021). Older patients with lung cancer need help with sleeping especially if in pain, especially in elderly patients with lower pain threshold levels. Therefore, sedative hypnotic drugs are taken more frequently than elderly patients without lung cancer. In order to ensure the risk-benefit balance of drug use in this population, it is of great significance to develop models that meet the PK/PD characteristics of this population to evaluate the appropriate medication use rather than keeping to a specific number of medicines in this fragile population. According to the logistic regression analysis, risk factors for PIM use were the same among the three models: 75–79 years of age, polypharmacy, irrational use of drugs and lung cancer accompanied by sleep disorders, anxiety or depression, or pain. However, some pulmonary diseases, such as pulmonary infection and COPD, were not risk factors for PIM use. Therefore, we suggest reducing the prescription of unnecessary medications and that doctors or pharmacists carefully perform medication reconciliation for older lung cancer outpatients taking multiple medications.

Several limitations of this study should be noted. First, this study only investigated 3 years of data in China, and more years of data are needed to determine long-term trends of polypharmacy and PIM use in older lung cancer outpatients with multimorbidity. Second, outpatients attending tertiary hospitals were the main focus of the study; thus, lung cancer outpatients who were in nursing homes and communities were not evaluated. In addition, the research aimed at only one area population may have limited popularization.

## Conclusion

This study investigated the trends of polypharmacy and PIM use in older lung cancer outpatients with multimorbidity in China based on the 2019 Beers criteria. The prevalence of polypharmacy and PIM use showed an increasing trend in older Chinese lung cancer outpatients, and age 75–79, polypharmacy, irrational use of drugs, and lung cancer accompanied by sleep disorders, anxiety or depression, or pain were risk factors for PIM use.

## Data Availability

The original contributions presented in the study are included in the article/Supplementary Materials; further inquiries can be directed to the corresponding author.
