# Supplementary material for: Increasing Trends of Polypharmacy and Potentially Inappropriate Medication Use in Older Lung Cancer Patients in China: A Repeated Cross-Sectional Study
Source: Front Pharmacol. 2022 Jul 18;13:935764. doi: 10.3389/fphar.2022.935764 (PMC9340379; doi:10.3389/fphar.2022.935764)
Supplement: Supplementary file 1 [file Table1.DOCX]

**Supplemental material: List of medications**

| **Sleep disorders** | **Anxiety or depression** | **Pain** | **Pulmonary infection** | **COPD** |
| --- | --- | --- | --- | --- |
| Estazolam  Alprazolam  Clonazepam  Diazepam  Zolpidem  Zopiclone | Escitalopram  Flupentixol / melitracen  Sertraline  Venlafaxine  Tandospirone  Mirtazapine  Paroxetine  Duloxetine  Fluoxetine  Amitriptyline  Estazolam  Alprazolam  Zolpidem | Morphine  Tramadol  Oxycodone  Fentanyl  Aminophenol oxycodone  Aminophenol tramadol  Spray him Zosin  Gabapentin  Naproxen  Lofentine  Meloxicam  Loxoprofen  Nimesulide  Celecoxib  Pregabalin  Arecoxib  Buprofen  Diclofenac sodium | Piperacillin sodium / sulbactam sodium  Cefoperazone sodium / tazobactam sodium  Cefaclor  Ceftriaxone  Cefdinir  Amoxicillin  Cefradine  Cefoxime  Cefpropene  Levofloxacin  Moxifloxacin  Roxithromycin  Clarithromycin | Salmeterol-Fluticasone  Budesonide/ Formoterol  Tiotropium bromide  Salbutamol  Terbutaline  Prednisone  Methylprednisolone  Ambroxol  Theophylline  Doxofylline  Aminophylline  Montelukast  Fordosteine  Carbocysteine  Acetylcysteine |

COPD, chronic obstructive pulmonary disease
